# Serum MicroRNA-155 in Acute Graft-Versus-Host-Disease (aGVHD)

**DOI:** 10.29328/journal.ijbmr.1001007

**Published:** 2019-08-16

**Authors:** Yvonne A Efebera, Amy S Ruppert, Apollinaire Ngankeu, Sabrina Garman, Prasanthi Kumchala, Alan Howard, Steven M Devine, Parvathi Ranganathan, Ramiro Garzon

**Affiliations:** 1 Division of Hematology, Department of Internal Medicine and Comprehensive Cancer Center, The Ohio State University, Columbus, Ohio, USA; 2 Blood and Marrow Transplant Clinical Trial Network, National Marrow Donor Program, USA

**Keywords:** Mir-155, Graft versus host disease, MicroRNA, Allogeneic stem cell transplant

Allogeneic hematopoietic stem cell transplant (alloHSCT) is a curative treatment for many hematologic malignancies. Unfortunately, about 30–50% of all recipients undergoing alloHSCT develop acute graft-versus-host-disease (aGVHD), which is associated with high morbidity and mortality [1,2]. Treatment of aGVHD involves the use of immune suppressive drugs such as high dose of steroids that leads to further immunosuppression and risk for opportunistic infections. Often patients are refractory to steroids therapy making the prognosis dismal. Thus, it is critical to identify robust biomarkers to detect aGVHD before onset of clinical symptoms so that therapeutic strategies can be implemented that may result in better treatment responses and less toxicity.

Over the past few years, several plasma proteins have been identified and validated to predict aGVHD occurrence, severity and treatment response. These plasma biomarkers include among others Interleukin 2 receptor, tumor necrosis alpha receptor 1, interleukin 8, hepatocyte growth factor and receptor suppression of tumorigenicity 2 (ST2) [3–7]. These biomarkers are now recognized as robust and reliable by experts in the field [8,9]. However, protein-based assays such as ELISA and mass spectrometry have drawbacks such as the need for high volume and high affinity quality antibodies with the required affinity and specificity for the target [9]. Further, focusing only on proteins may not be enough to develop powerful and comprehensive biomarkers. Thus, it will be important to investigate whether cell free DNA or RNA that are detectable in the plasma or serum predict aGVHD and provide prognostic information and whether these biomarkers may complement the current validated protein-based tests.

In the past years, circulating microRNAs (miRs) have emerged as possible candidates for aGVHD biomarkers. A panel of four miRs (miR-423, miR-199a-3p, miR-93*, and miR- 377) were found to be over-expressed in the plasma of aGVHD patients 16 days before diagnosis as compared to non-GVHD patients [10]. One study showed that plasma miR-586 was upregulated in patients with aGVHD [11], and 2 independent studies identified miR-155 to be upregulated in the serum [12] and whole blood [13] of patients that developed aGVHD versus those who did not. Since miR-155 expression in T cells and in antigen presenting cells (APCs) is critical for aGVHD pathogenesis [14,15], we sought to validate the previous reports on high levels of circulating miR-155 in aGVHD. Thus, to investigate whether serum miR-155 was elevated in patients at time of aGVHD onset following alloHSCT as compared to matched controls, we measured miR-155 expression from serum samples obtained from patients enrolled in the Blood and Marrow Transplant Clinical Trial Network (BMT CTN) 0101 study, a randomized, double-blind trial of fluconazole vs. voriconazole for prevention of invasive fungal infection after alloHSCT [16]. From 98 patients enrolled on this trial, samples at aGVHD onset within 100 days from alloHSCT were matched with controls without aGVHD (n=49 pairs). Patient samples were matched on day from alloHSCT (+/− 7 days in 96% of pairs), histology of disease (acute leukemia and MDS vs. CML vs. lymphoma), receipt of GVHD prophylaxis, and type of donor (related vs. unrelated). The samples were obtained from the National Heart, Lung, and Blood Institute Biologic Specimen Repository (NHLBI Biorepository).

Serum total RNA was extracted using the miRNeasy Serum/Plasma Kit (Cat. no.217184, QIAGEN, Valencia, CA, USA) with a modified protocol. 200 μL of serum sample was carefully transferred to a new 2-mL DNA-LoBind tube (Eppendorf, Hamburg, Germany). Serum was mixed with 1 ml QIAzol Lysis Reagent. The mixture was placed on a vortex mixer at 3000 rpm for 30 s and then left at room temperature for 5 min to allow complete inactivation of serum RNases. 4 μL of a mixture of serial dilution (1, 0.1 and 0.01 pg/ μL) of pooled synthetic microRNA was spiked into the homogenate lysis mixture. From here, the manufacturer’s protocols were followed for RNA extraction (Cat. no.217184, QIAGEN, Valencia, CA, USA). Qiagen miScript PCR System was used for reverse transcription and RT-qPCR. 100ng of RNA was converted into cDNA using the miScript II Reverse Transcription Kit (Cat. N.218161) and with the HiSpec Buffer according to the manufacturer’s protocol. The RT-qPCR was performed with the miScript SYBR® Green PCR Kit (Cat. N. 218073) in a total volume of 20μl per reaction containing 2μl diluted cDNA according to the manufacturer’s protocol. C-elegans microRNAs spikein control expression was used for normalization. The miScript universal primer and the miRNA-specific miScript primer assay for Hs_miR-155 (Cat. MS00031486), Ce_miR-238 (Cat. MS00019439), Ce_miR-54 (Cat. MS00019894) and Ce_miR-39 (Cat. 219610) (all from QIAGEN), were used to detect mature miRNA. Individual real-time PCR assays were performed in triplicate wells on an ABI 7500 real-time PCR system (Applied Biosystems) and results were averaged. A paired t-test was used to compare -ΔCT values for serum miR-155 expression between aGVHD patients and non-aGVHD controls. Conditional logistic regression was used to model the probability of being called the case within a matched pair as a function of miR-expression and important or confounding variables not accounted for by the matching. All tests were two-sided and statistical significance was declared for p<0.05.

Patient characteristics are provided in table 1. Among the 49 cases, 39 had grade 2 aGVHD and 10 had grade 3 aGVHD at onset. The median time to aGVHD onset was 21 days from transplant (range: 4 – 91). We found no significant difference in miR-155 expression between aGVHD cases and matched controls (p=0.16; Figure 1). Although expression was higher in 31 cases (63%), the average -ΔCT was higher by 0.29 (95% CI: −0.12 to 0.70) and corresponded to a non-significant relative fold change of 1.22 (95% CI: 0.92 to 1.63). The only clinical variable significantly associated with aGVHD was donor source, with 84% of cases receiving stem cell source from peripheral blood compared to 61% of controls (p=0.02; Table 1). When adjusting for donor source, the association between miR-155 and presence of aGVHD remained non-significant (p=0.16).

Hence, no significant difference in miR-155 expression was observed in the serum of patients at onset of aGVHD compared to those who did not have aGVHD. This is in contrast to previous reports showing an upregulation of serum and whole blood miR-155 in aGVHD [12,13]. Possible reasons for this discrepancy could be 1: Sample size and patient population: The sample size of the previous studies that assessed mir-155 association with aGVHD are small (N=25–28) [12,13]. While our study is also small, it is almost twice as large and had at least 80% power to detect a relative fold change in miR-155 expression of 1.54 when the standard deviation of the differences in -ΔCT values between cases and controls was as large as 1.5, as was observed in our dataset. The patient population studied in the previous studies were wide, including non-hematologic disease, while this study focused on hematologic malignancies with 92% being leukemias and myelodysplasia. 2: Timing of assessment of samples: The clinical trial from which patient samples were obtained was conducted between 2003 and 2006 and samples were stored in the NHLBI biorepository. It is therefore possible that some degradation may have occurred. It is however noteworthy that there is little overlap in the circulating miRNAs identified as prognostic by profiling studies, which could be explained by the different sources used (plasma vs. serum), profiling methods and small numbers of patients. Despite these findings, this in no way rules out a role for microRNAs in the pathogenesis of acute GVHD, but at the moment they do not serve as reliable biomarkers. We realize the limitations of this study: lack of samples from peak aGVHD and lack of serial samples that precludes evaluation of miR-155 at a time point prior to aGVHD onset or change in miR-155 expression as a prognostic factor. There is therefore the need to conduct comprehensive studies with serial samples obtained from a sufficiently large number of patients with robust profiling methods to derive reliable RNA or DNA circulating biomarkers.

## Figures and Tables

**Figure 1:**
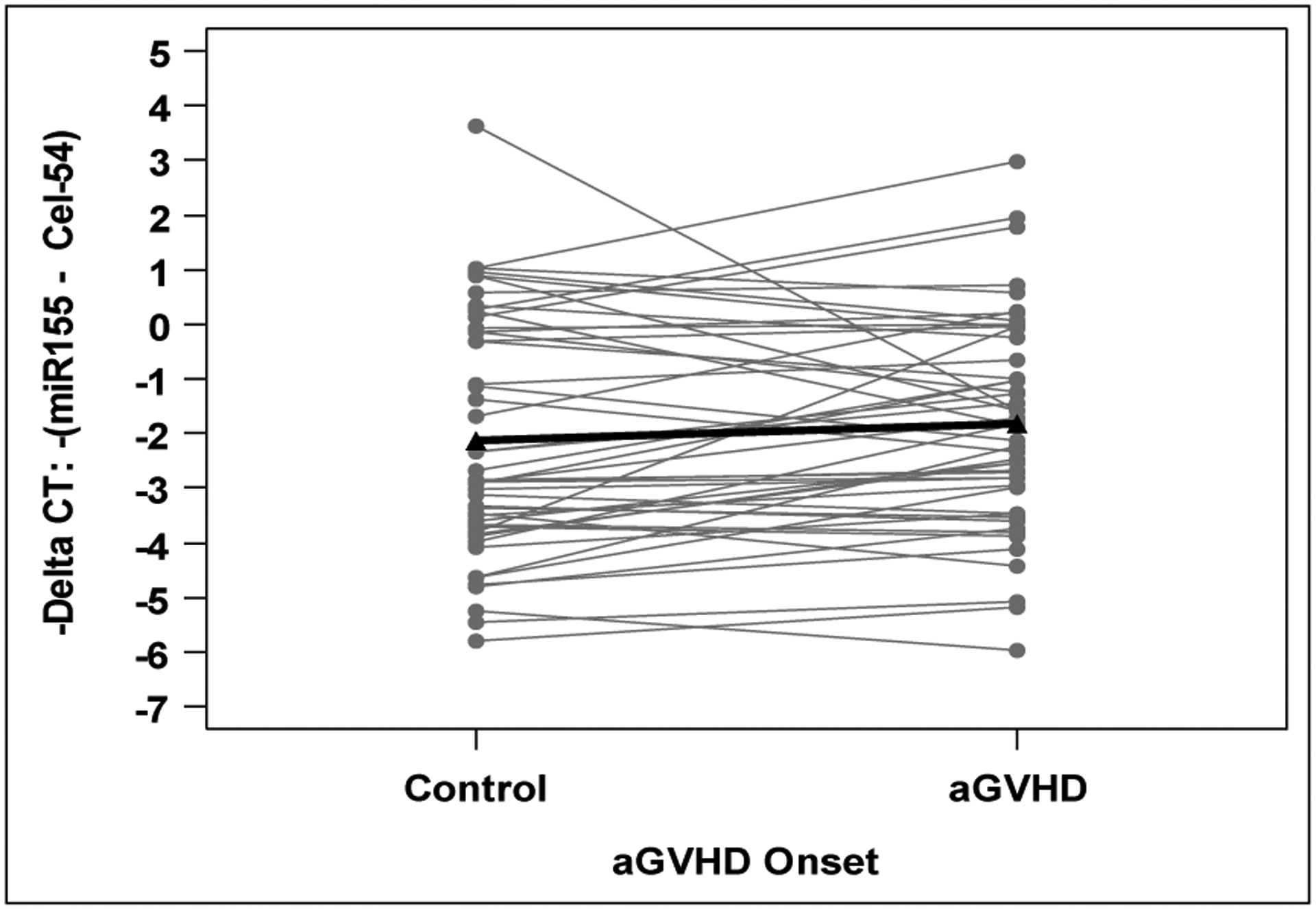
miR-155 expression is depicted as -ΔCT values, with higher values corresponding to higher expression. Using a paired t-test, the -ΔCT was on average 0.29 units higher (95% CI: −0.12 to 0.70) in cases compared to matched controls (p = 0.16). This corresponds to a non-significant relative fold change of 1.22 (95% CI: 0.92 to 1.63) in cases versus matched controls.

**Table 1:** Patient Characteristics.

Characteristic	All Patients n=98	Case n=49	Controls n=49	p-value*
Patient age in years at transplant, no. (%)				
≤30	27 (28)	12 (24)	15 (31)	0.30
31–50	49 (50)	23 (47)	26 (53)	
>50	22 (22)	14 (29)	8 (16)	
Patient sex, no. (%)				
Male	49 (50)	22 (45)	27 (55)	0.32
Females	49 (50)	27 (55)	22 (45)	
Patient race, no. (%)				
White	87 (92)	44 (90)	43 (93)	0.42
Non-white	8 (8)	5 (10)	3 (7)	
Unknown	3	0	3	
Disease diagnosis, no. (%)				
Acute Leukemia/MDS	78 (80)	39 (80)	39 (80)	NA
CML	12 (12)	6 (12)	6 (12)	
Lymphoma	8 (8)	4 (8)	4 (8)	
Performance score, no. (%)				
100	32 (33)	15 (31)	17 (35)	0.66
90	56 (57ss)	30 (61)	26 (53)	
≤80	10 (10)	4 (8)	6 (12)	
Prophylaxis treatment, no. (%)				
Fluconazole	48 (49)	24 (49)	24 (49)	NA
Voriconazole	50 (51)	25 (51)	25 (51)	
HLA score, no. (%)				
5/6	2 (2)	1 (2)	1 (2)	1.00
6/6	96 (98)	48 (98)	48 (98)	
Stem cell source				
Marrow	27 (28)	8 (16)	19 (39)	0.02
PBSC	71 (72)	41 (84)	30 (61)	
Donor relationship, no. (%)				
Related	64 (65)	32 (65)	32 (65)	NA
Unrelated	34 (35)	17 (35)	18 (35)	

**Abbreviations**:MDS=myelodysplastic syndrome; CML=chronic myelogenous leukemia; PBSC=peripheral blood stem cell; NA=not applicable; P-values from univariable conditional logistic regression models that account for the individual matching of cases to controls based on disease diagnosis, prophylaxis treatment, and donor relationship are presented. Models cannot be fit for the variables upon which the matching was performed and thus p-values are not applicable.
